# Associations between metabolic syndrome and erectile dysfunction: evidence from the NHANES 2001–2004

**DOI:** 10.3389/fpubh.2025.1543668

**Published:** 2025-03-18

**Authors:** Wei Wang, Shuai Zhao, Ran Zhou, Pei-Ze Yu, Si-Yuan Pan, Peng-Fei Huan, Zhen-Duo Shi, Ying Liu, Xiao Hu, Jing-Ru Lu, Conghui Han

**Affiliations:** ^1^School of Medicine, Southeast University, Nanjing, China; ^2^Department of Urology, Xuzhou Central Hospital, Xuzhou, China; ^3^Department of Urology, ZiBo 148 Hospital, China RongTong Medical Healthcare Group Co. Ltd., Zibo, China; ^4^School of Medicine, Xuzhou Medical University, Xuzhou, China; ^5^Department of Central Laboratory, Xuzhou Central Hospital, Xuzhou, China; ^6^Department of Nephrology, Shandong Provincial Hospital Affiliated to Shandong First Medical University, Jinan, China

**Keywords:** metabolic syndrome, erectile dysfunction, NHANES, prevalence, cross-sectional study

## Abstract

**Background and objectives:**

Erectile dysfunction is a common clinical condition that seriously affects the quality of life and mental health of men and their partners. Metabolic syndrome (MetS) is the most important public health problem threatening men’s health worldwide, and its current prevalence continues to grow. This study examines the relationship between metabolic syndrome and erectile dysfunction (ED).

**Method:**

We conducted a cross-sectional study with data were sourced from NHANES 2001–2004. In this study, the relationship between METS-VF and ED was analyzed using multivariate logistic regression, followed by subgroup analyses to identify sensitive populations. Comparative logistic regression of the Receiver Operating Characteristic (ROC) curve assessed the diagnostic capability of METS-VF against the classical obesity index for ED. Creating Predictive Histograms for ED Patients and assess the net benefit of the model through Decision Curve Analysis (DCA).

**Results:**

The study enrolled 1,374 participants, of whom 545 self-reported ED history. There was a significant positive association between metabolic syndrome and erectile dysfunction (ED). The risk of ED in people with metabolic syndrome was 2.32 times higher than that in people without metabolic syndrome (dominance ratio = 2.32, 95% confidence interval: 1.83–2.96, *p* < 0.001). Subgroup analysis highlighted a stronger correlation in participants aged 50–85 years, hypertensive individuals, and those with large belly circumference. A histogram model including three variables: metabolic syndrome, age and smoking status was constructed to predict the probability of ED occurrence. And decision curve analysis (DCA) was used to assess the net benefit of its nomogram model at different high-risk thresholds. The high clinical utility of the model under different thresholds was illustrated.

**Conclusion:**

The risk of ED in people with metabolic syndrome was 2.32 times higher than that in people without metabolic syndrome. Furthermore, this observed positive correlation emphasizes the need for increased vigilance in patients with advanced age, smoking, and MetS.

## 1 Introduction

Metabolic syndrome (MetS) is a group of disorders associated with the development of cardiovascular disease and type 2 diabetes mellitus, characterized by elevated blood pressure, centripetal obesity, hyperglycemia, hypertriglyceridemia, and low high-density lipoprotein cholesterol (HDL) cholesterol (1). In the United States, the prevalence of MetS can be as high as 35–39% (2). Previous studies have found that the MetS is associated with increased cardiovascular disease, hypertension, stroke, and all-cause mortality (3, 4). Thompson et al. (5) found a correlation between elevated biomarkers of inflammation and endothelial dysfunction and increased odds of pre-diabetes, diabetes mellitus and MetS among Chinese adults. Endothelial dysfunction can lead to vasculopathy in ED. With economic development, the prevalence of metabolic syndrome is increasing year by year throughout the world, imposing a serious economic burden on society (6, 7). Different classifications have been developed to define MetS, but it is worth noting that recent studies have linked MetS to erectile dysfunction (ED) (8, 9).

Erectile dysfunction is a very common clinical condition that seriously affects the quality of life of men all over the world. According to statistics, the prevalence of ED is as high as 52% among male patients over 40 years of age and increases with age. By 2025, the number of people suffering from ED will reach 322 million worldwide (10). The Massachusetts Male Aging Study (MMAS) showed that the overall probability of complete ED in men at age 40 was 5% and increased to 15% by age 70 (11). In a survey of ED in men aged 40–79 years in eight European centers, the prevalence of ED was higher in older adults and peaked at age 70 years (12). The etiology of ED is not a single factor but is caused by a combination of factors, such as neurological, vascular and hormonal, etc. Hypertension, diabetes, hypercholesterolemia, and many other factors are part of MetS and may all be risk factors for ED (13).

Therefore, we conducted an American population-based cross-sectional study using a large sample from the National Health and Nutrition Examination Survey (NHANES) to explore the correlation between MetS and ED among adult men in the U.S. and to further determine how valuable MetS is in predicting ED.

## 2 Materials and methods

### 2.1 Study population

NHANES is a well-designed study conducted by the National Center for Health Statistics (NCHS) at multiple sites. The purpose of the study was to investigate the nutrition and health of the general population in the United States (14). NHANES conducts a comprehensive cross-sectional survey every 2 years, with participants representing approximately 50,000 U.S. citizens at a time, and is approved by the U.S. Centers for Disease Control and Prevention (CDC) Institutional Review Board. For detailed data and information on NHANES, please visit https://www.cdc.gov/nchs/nhanes/index.htm.

**Figure 1:**
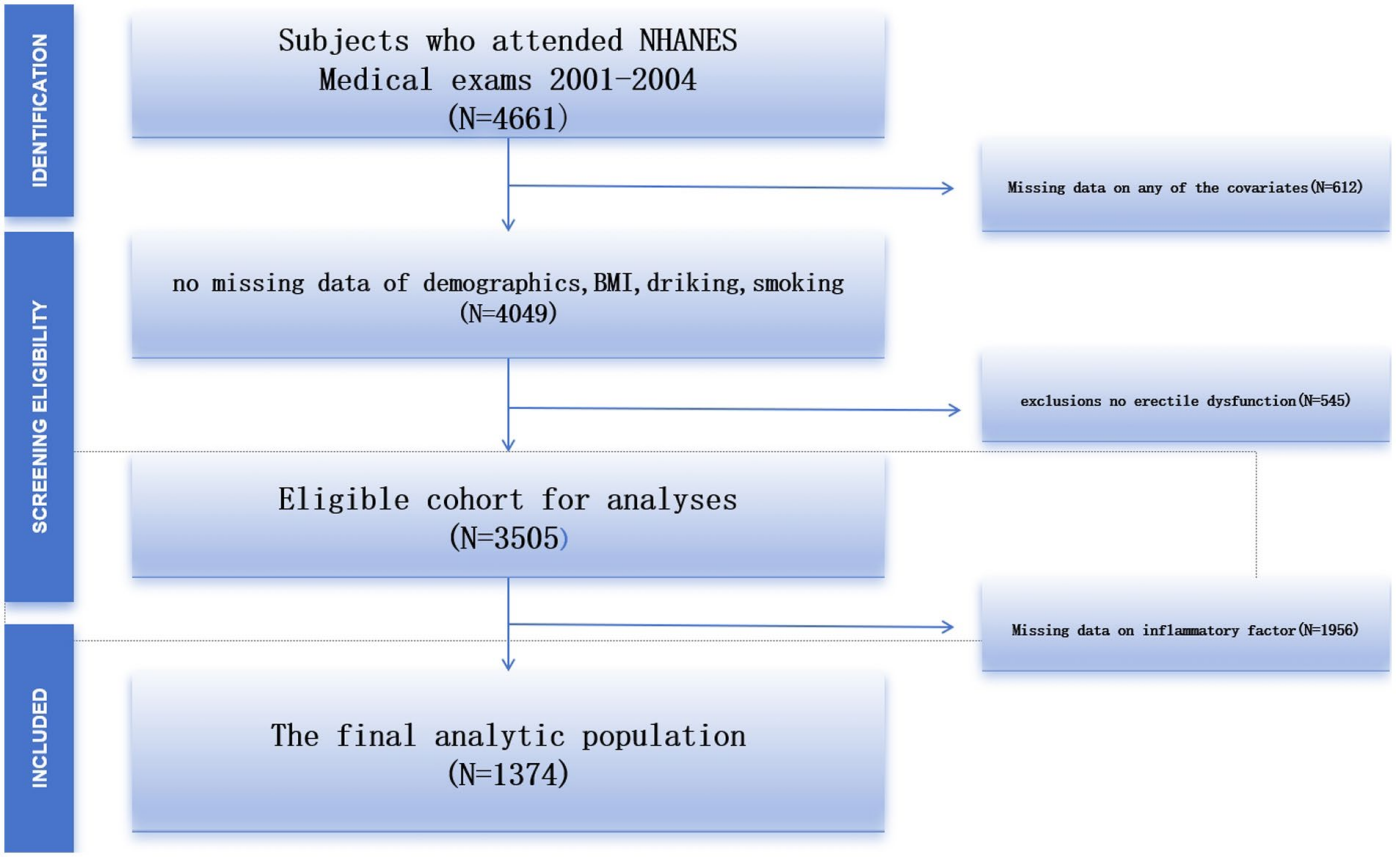
Flowchart of participant selection.

In this cross-sectional study, we focused primarily on the 2001–2004 data because the ED questionnaire was available only from 2001 to 2004. The questionnaire was administered only to adult males over the age of 20 years and adult males and females under the age of 20 years were excluded from the data and a total of 4,661 subjects were included. After excluding 612 subjects with missing data, 545 subjects without erectile dysfunction, and 1956 subjects with missing data on inflammatory factors, the study ultimately included 1,374 eligible subjects (Figure 1).

### 2.2 Exposure variables

In this study, the exposure variables were the MetS and its components, consisting of High blood glucose, high blood pressure, high triglycerides, low high-density lipoprotein cholesterol, and cardiovascular obesity. On the basis of the National Cholesterol Education Program (NCEP) Adult Treatment Panel III (ATPIII) (15), MetS as a combination of at least three out of five of the following: (1) hyperglycemia that is ≥100 mg/L; (2) hypertension as systolic blood pressure ≥ 130 and/or diastolic blood pressure ≥ 85 mmHg; (3) hyperglycemia (≥100 mg/L); (4) low high density lipoprotein cholesterol ranges are <40 mg/L in men and <50 mg/L in women; (5) (1) Obesity is defined as an increase in waist circumference of ≥102 centimeters in men and ≥88 centimeters in women. Waist circumference is a measurement of the distance between the front and back of the abdomen and the horizontal line of the upper lateral margins of the iliac bones on each side. Blood pressure was measured three times in a row after a five-minute break. If there is an interruption in the blood pressure measurement, a fourth measurement may be taken. In this study, glucose, cholesterol and total cholesterol were examined by drawing venous blood on an empty stomach.

### 2.3 Outcome variable

Erectile dysfunction was the outcome variable of the study. Self-Interview (ACASI) utilizing computer-assisted audio in a private room. ED was self-assessed using a question from the Massachusetts Male Aging Study (16, 17).

### 2.4 Handling of missing values

Continuous variables with many missing values are converted to categorical variables, and the missing variables are set as a dummy variable group and named “unclear.”

### 2.5 Statistical methods

Each statistical process was analyzed using appropriate NHANES sampling weights and taking into account the complex multi-stage cluster survey design. The study population’s baseline characteristics table was divided into two groups based on whether participants had ED. The study population characteristics were also stratified according to participants’ gender, age, and race/ethnicity. Recurring variables are expressed as mean ± SD, and categorical variables are expressed as percentages. Both weighted linear regression and weighted chi-square tests were used to compare differences between continuous and categorical variables at baseline, respectively. We built three different multiple regression models using MetS and ED. Model 1 does not include adjustment for covariates. Model 2 adjusts for race, education level, and marital status. Model 3 contains adjustments for all variables. We used a generalized additive model (GAM) and smoothed curve fitting to examine the relationship between MetS and ED and the points of influence. We used a two-stage linear regression model to fit each interval and quantify threshold effects if non-linear patterns emerged. At last, WWI, BMI and WC were evaluated for their predictive effects on ED by receiver operating characteristic curve (ROC) and area under the curve (AUC) calculations. We performed all statistical analyses using R (version 4.2.0) and EmpowerStats (version 2.0). *p* < 0.05 was statistically significant.

## 3 Results

### 3.1 Participant characteristics

A total of 1,374 survey individuals aged from 20 to 85 years were enrolled in our study. The mean age of participants was 49.40 ± 18.19 years, of whom 22.20% were Mexican American, 55.31% were non-Hispanic white, 16.96% were non-Hispanic black, 2.84% were other Hispanic, and 2.69% were from other races. The mean BMI and Height were 27.78 ± 5.01 (kg/m^2^) and 175.10 ± 7.86 (cm).

Three hundred and seventy-nine of 1,374 (27.58%) individuals had a history of ED. Among participants in the ED group, there were significantly more patients with concomitant MetS than those without [225 vs. 154]. Men with ED were likely to be older, lower in height, less educated, former smokers, have wider waist circumference, be hypertensive, and have diabetes mellitus (all *p* < 0.05). The detailed demographic data of all survey individuals are demonstrated in Table 1.

**Table 1:** Baseline characteristics of two groups.

Variables	Overall (*n* = 1,374)	Non-ED (*n* = 995, 72.42%)	ED (*n* = 379, 27.58%)	*p*-value
Age, years, mean (SD)	49.40 ± 18.19	43.38 ± 15.48	65.19 ± 15.07	<0.001
Weight, cm, mean (SD)	85.39 ± 17.43	85.68 ± 17.17	84.64 ± 18.12	0.335
Height, cm, mean (SD)	175.10 ± 7.86	175.81 ± 7.90	173.21 ± 7.46	<0.001
BMI, kg/m^2^, mean (SD)	27.78 ± 5.01	27.66 ± 4.95	28.11 ± 5.15	0.146
Race, *n* (%)				0.002
Mexican American	305 (22.20%)	217 (21.81%)	88 (23.22%)	
Other Hispanic	39 (2.84%)	28 (2.81%)	11 (2.90%)	
Non-Hispanic White	760 (55.31%)	528 (53.07%)	232 (61.21%)	
Non-Hispanic Black	233 (16.96%)	190 (19.10%)	43 (11.35%)	
Other race, including multi-racial	37 (2.69%)	32 (3.22%)	5 (1.32%)	
Education, *n* (%)				<0.001
Less than 11th grade	385 (28.02%)	233 (23.42%)	152 (40.11%)	
High school or GED	322 (23.44%)	254 (25.53%)	68 (17.94%)	
Some college or AA degree above	667 (48.54%)	508 (51.06%)	159 (41.95%)	
Marital status, *n* (%)				0.003
Married/Living with partner	959 (69.80%)	671 (67.44%)	288 (75.99%)	
Living alone	415 (30.20%)	324 (32.56%)	91 (24.01%)	
Family PIR (%)				0.096
<1.5	356 (25.91%)	250 (25.13%)	106 (27.97%)	
1.5–3.5	487 (35.44%)	343 (34.47%)	144 (37.99%)	
≥3.5	531 (38.65%)	402 (40.40%)	129 (34.04%)	
Alcohol user, *n* (%)				0.323
No	832 (60.55%)	594 (59.70%)	238 (62.80%)	
Yes	542 (39.45%)	401 (40.30%)	141 (37.20%)	
Smoking status, *n* (%)				<0.001
No	515 (37.48%)	429 (43.12%)	86 (22.69%)	
Yes	859 (62.52%)	566 (56.88%)	293 (77.31%)	
Waist Circumference, *n* (%)				<0.001
≤90	339 (24.67%)	280 (28.14%)	59 (15.57%)	
>90	1,035 (75.33%)	715 (71.86%)	320 (84.43%)	
Systolic pressure, *n* (%)				<0.001
<130	857 (62.37%)	685 (68.84%)	172 (45.38%)	
≥130	517 (37.63%)	310 (31.16%)	207 (54.62%)	
Diastolic pressure, *n* (%)				0.393
<85	1,169 (85.08%)	841 (84.52%)	328 (86.54%)	
≥85	205 (14.92%)	154 (15.48%)	51 (13.46%)	
Fasting blood-glucose, *n* (%)				<0.001
<100	738 (53.71%)	602 (60.50%)	136 (35.88%)	
≥100	636 (46.29%)	393 (39.50%)	243 (64.12%)	
Triglyceride, *n* (%)				0.027
≤150	913 (66.45%)	679 (68.24%)	234 (61.74%)	
>150	461 (33.55%)	316 (31.76%)	145 (38.26%)	
HDL-cholesterol, *n* (%)				0.263
<40	324 (23.58%)	243 (24.42%)	81 (21.37%)	
≥40	1,050 (76.42%)	752 (75.58%)	298 (78.63%)	
Metabolic syndrome, *n* (%)				<0.001
No	765 (55.68%)	611 (61.41%)	154 (40.63%)	
Yes	609 (44.32%)	384 (38.59%)	225 (59.37%)	
Laboratory features				
Waist circumference, cm, mean (SD)	99.34 ± 13.90	97.86 ± 13.58	103.23 ± 14.01	<0.001
Systolic pressure, mmHg, mean (SD)	126.89 ± 19.11	124.33 ± 17.14	133.60 ± 22.16	<0.001
Diastolic pressure, mmHg, mean (SD)	72.46 ± 13.64	73.43 ± 12.87	69.89 ± 15.22	<0.001
Fasting blood-glucose, mg/dL, mean (SD)	105.76 ± 29.75	101.67 ± 22.00	116.50 ± 42.21	<0.001
Triglyceride, mg/dL, mean (SD)	137.04 ± 71.08	133.31 ± 69.88	146.83 ± 73.31	0.002
HDL-cholesterol, mg/dL, mean (SD)	48.32 ± 12.66	48.37 ± 12.69	48.19 ± 12.61	0.807

### 3.2 The association between MetS and ED

Weighted univariable logistic regression was conducted to assess the association of MetS and all chosen covariates with ED. Detailed information was shown in Table 2. According to the results of univariate logistic regression analysis in Table 2, there was a significant positive association between metabolic syndrome and erectile dysfunction (ED). The risk of ED in people with metabolic syndrome was 2.32 times higher than that in people without metabolic syndrome (dominance ratio = 2.32, 95% confidence interval: 1.83–2.96, *p* < 0.001). This suggests that metabolic syndrome may be an important risk factor for ED.

**Table 2:** The univariate logistic regression analysis.

Variables	OR (95%CI)	*P*
Age, years, mean (SD)	1.09 (1.08, 1.10)	<0.001
Weight, cm, mean (SD)	1.00 (0.99, 1.00)	0.323
Height, cm, mean (SD)	0.96 (0.94, 0.97)	<0.001
BMI, kg/m^2^, mean (SD)	1.02 (0.99, 1.04)	0.139
Race, *n* (%)		
Mexican American	Reference	
Other Hispanic	0.98 (0.45, 2.01)	0.950
Non-Hispanic White	1.08 (0.81, 1.45)	0.593
Non-Hispanic Black	0.56 (0.37, 0.84)	0.005
Other race, including multi-racial	0.40 (0.13, 0.97)	0.042
Education, *n* (%)		
Less than 11th grade	Reference	
High school or GED	0.41 (0.29, 0.57)	<0.001
Some college or AA degree above	0.48 (0.37, 0.63)	<0.001
Marital status, *n* (%)		
Married/Living with partner	Reference	
Living alone	0.66 (0.50, 0.86)	0.002
Family PIR (%)		
<1.5	Reference	
1.5–3.5	0.99 (0.73, 1.34)	0.947
≥3.5	0.76 (0.56, 1.02)	0.071
Alcohol user, *n* (%)		
No	Reference	
Yes	0.88 (0.69, 1.12)	0.294
Smoking status, *n* (%)		
No	Reference	
Yes	2.58 (1.97, 3.40)	<0.001
Metabolic syndrome, *n* (%)		
No	Reference	
Yes	2.32 (1.83, 2.96)	<0.001

In the overall population, one-way logistic regression showed that MetS was positively associated with ED (OR: 2.32, 95% CI: 1.83, 2.96). This association persisted after adjusting for age, BMI, and race in Model 2 (OR: 1.69, 95% CI: 1.29, 2.22). In model 3, after adjusting for all covariates, MetS remained significantly associated with ED incidence (OR: 1.61, 95% CI: 1.22, 2.14). The detailed results are shown in Table 3.

**Table 3:** The association between MetS levels and prevalence of ED by logistic regression analyses.

Variables	Model 1		Model 2		Model 3	
	OR (95%CI)	*P*-value	OR (95%CI)	*P*-value	OR (95%CI)	*P*-value
Metabolic syndrome						
No	1.00 (Reference)		1.00 (Reference)		1.00 (Reference)	
Yes	2.32 (1.83 ~ 2.96)	<0.001	1.69 (1.29 ~ 2.22)	<0.001	1.61 (1.22 ~ 2.14)	<0.001

OR, Odds Ratio; CI: Confidence Interval.

Model 1: Crude.

Model 2: Adjust: Age, BMI, Race.

Model 3: Adjust: Age, BMI, Race, Education, Marital status, Family PIR, Alcohol users, Smoking status.

**Figure 2:**
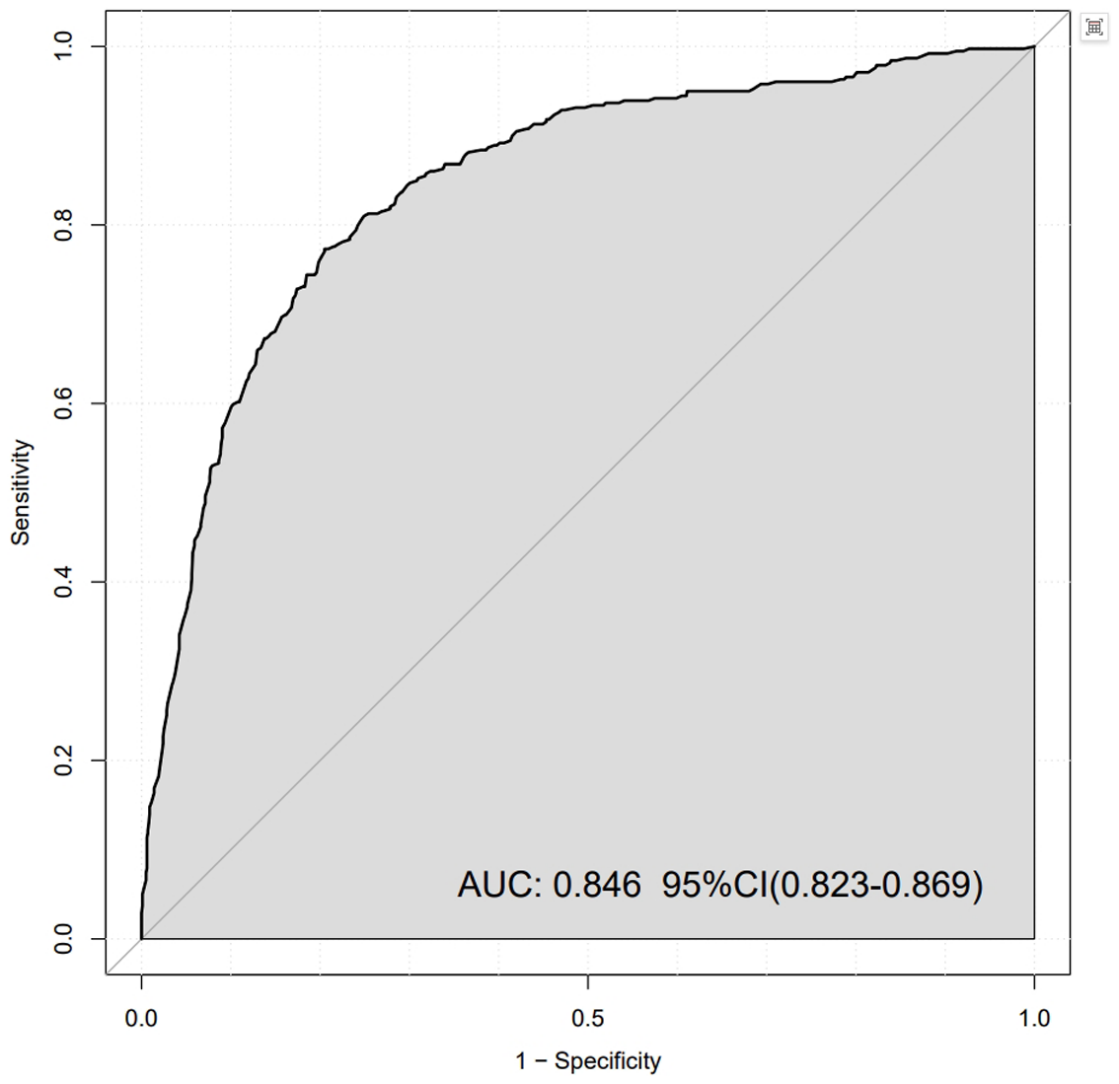
ROC curve for erectile dysfunction.

We plotted ROC curves to assess the prediction of ED by MetS (Figure 2). The horizontal axis represents ‘1 – specificity’ (false positive rate) and the vertical axis represents ‘sensitivity’ (true positive rate). The area under the curve (AUC) was 0.846, indicating good discriminatory power of the model. The 95% confidence interval of the AUC was 0.823 to 0.869, which means that the predictive performance of the model was considered statistically stable at a high level of confidence.

### 3.3 Subgroup analyses

**Figure 3:**
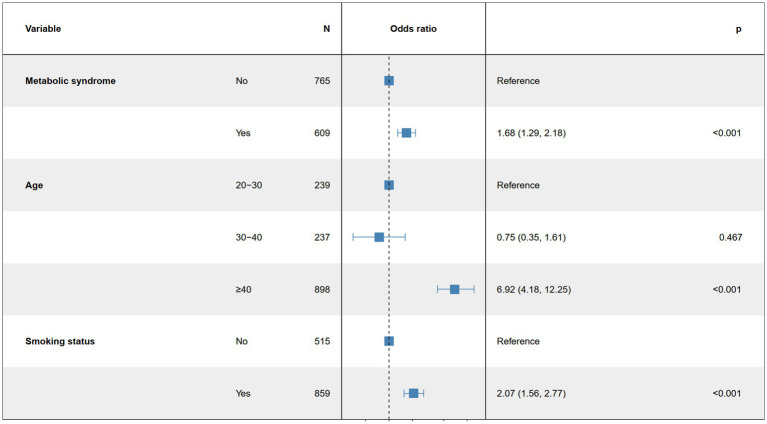
Multifactor logistic regression model.

Based on the univariate logistic regression analysis results in Table 2, we conducted a subgroup analysis to assess the robustness of the association between MetS and ED. In the age subgroup, we found that the authors overestimated the risk of ED in younger men and underestimated the risk of ED in older men in each group. For example, in our study, the relationship between MetS and the risk of ED was more pronounced over the age of 40 years. Increasing age was significantly associated with a higher risk of ED (OR = 1.09, 95% CI: 1.08, 1.10, *p* < 0.001). Smokers exhibited a significantly higher risk of ED (OR = 2.58, 95% CI: 1.97, 3.40, *p* < 0.001). Additionally, individuals with metabolic syndrome had a significantly increased risk of ED (OR = 2.32, 95% CI: 1.83, 2.96, *p* < 0.001). These results suggest that age, smoking status, and metabolic syndrome may play significant roles in the association between MetS and ED risk, indicating that these factors should be considered when evaluating related risks (Figure 3).

### 3.4 Creation of predictive column line graphs for ED patients

**Figure 4:**
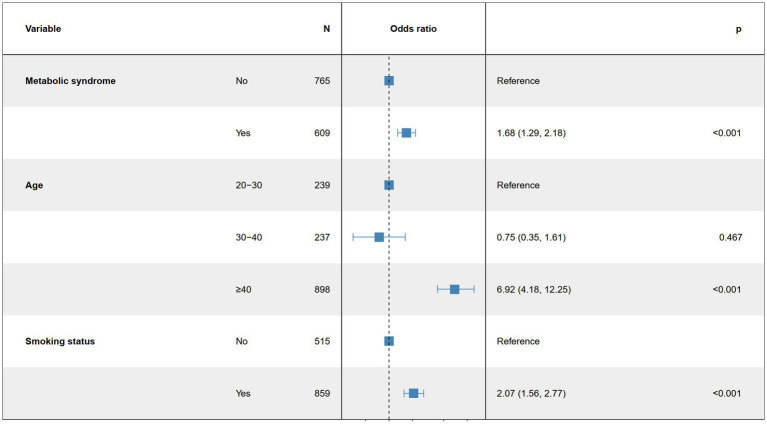
Predictive modelling.

This figure illustrates a nomogram model designed to predict the probability of ED (Figure 4). The model includes three variables: metabolic syndrome, age, and smoking status. Each variable is assigned a score based on its impact on the outcome. Metabolic syndrome and smoking status have two categories: “Yes” and “No,” while age ranges from 20 to 85 years. By summing the scores of each variable, a total score is obtained. This total score is then used to calculate the probability of the outcome event based on a function relating total score to predicted probability. The nomogram simplifies complex regression models into a visual format, allowing clinicians to quickly assess patient risk in practice.

**Figure 5:**
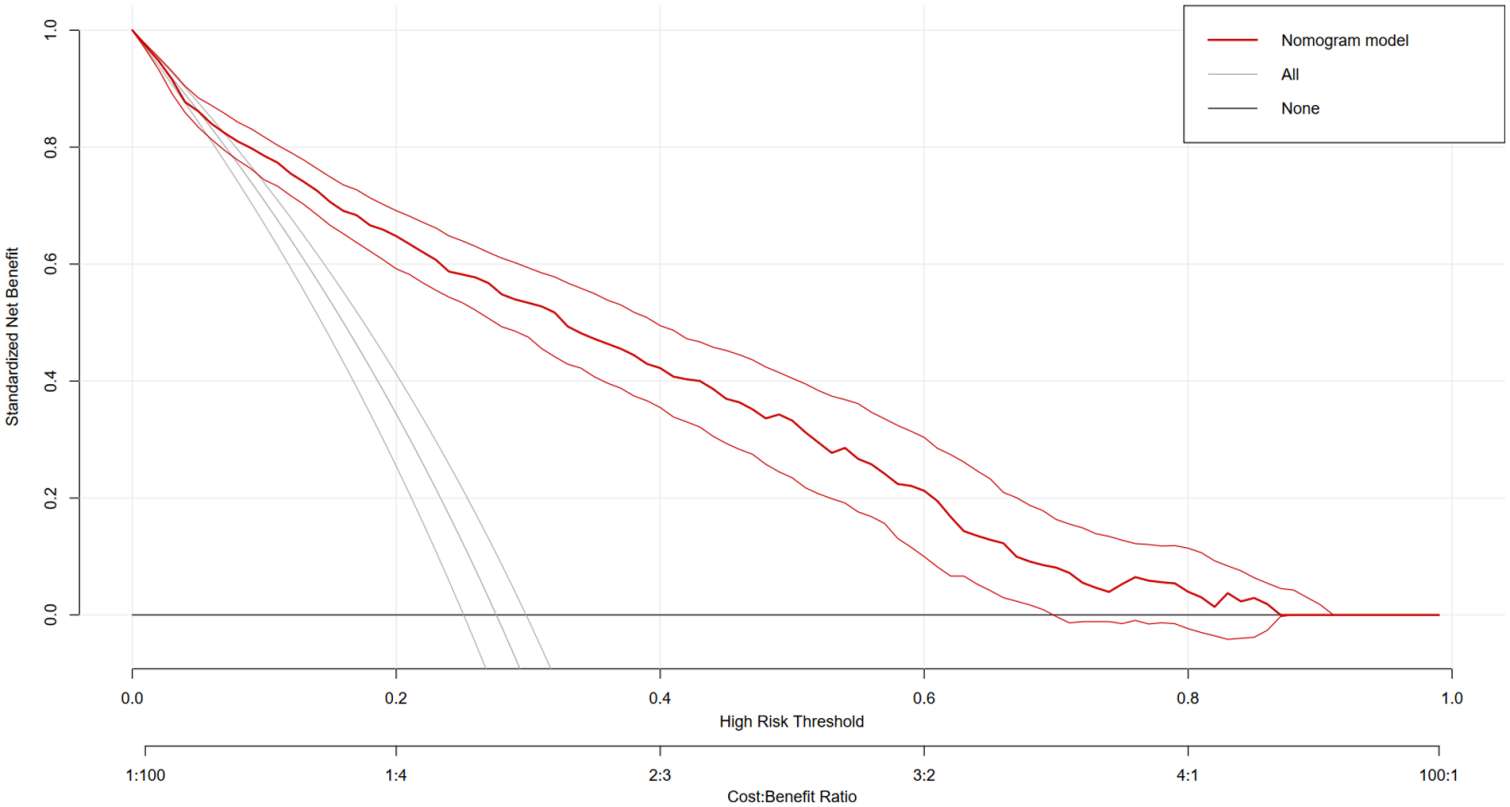
DCA evaluates the net benefit of the nomogram model across different high-risk thresholds.

Additionally, the decision curve analysis (DCA) evaluates the net benefit of the nomogram model across different high-risk thresholds (Figure 5). The red curve represents the nomogram model, while the gray and black curves represent the strategies of “assuming all individuals are at risk” and “assuming no individuals are at risk,” respectively. The vertical axis shows the standardized net benefit, and the horizontal axis represents the high-risk threshold, illustrating the model’s clinical utility at various thresholds. The nomogram model demonstrates higher net benefits over most high-risk thresholds, indicating superior clinical decision-making value at these levels.

## 4 Discussion

In this cross-sectional study, we explored the connection between MetS and ED in non-institutionalized U.S. residents and discovered that an elevated MetS was strongly linked with a higher likelihood of ED. Subgroup analyses showed that age, smoking status and metabolic syndrome may play an important role in the association between MetS and ED risk. In addition, we constructed a predictive model for three variables: metabolic syndrome, age, and smoking. When patients had metabolic syndrome, a history of smoking, and were older, they had a higher risk of ED, and DCA plots showed that the predictive model had a higher net benefit and a higher decision-making value in the clinical setting.

The association between MetS and ED has been supported by numerous studies. Heidler et al. (18) explored the correlation between MetS and ED in a total of 2,371 men and discovered that only in males ≥50 years old, a higher prevalence of MetS was related significantly to a greater risk ofED, and subjects with severe ED rose by 48%. García-Cruz et al. (19) showed that survey individuals with MetS had significantly lower scores on International Index of Erectile Function-5 (IIEF-5) (*p* < 0.001). Additionally, the incidence of moderate-to-severe ED was higher among participants with MetS than those without MetS (*p* < 0.001) (19). MetS is a pathological condition in which multiple metabolic components are abnormally aggregated, which is more harmful to health. Therefore, it is reasonable to speculate that MetS may have a positive correlation with ED prevalence.

Earlier research has ascertained that age, smoking, diabetes, sedentary behavior, cardiovascular disease and obesity exhibit robust correlations with ED development, with a subset of cases (20%) attributed to psychological factors (20, 21). MetS is a medical condition characterized by a combination of metabolic abnormalities, including insulin resistance, hyperglycemia, hyperlipidemia, hypertension, and obesity (22). Insulin resistance is linked to several negative consequences, such as endothelial dysfunction, reduced cardiac diastolic relaxation, impaired vascular relaxation, decreased coronary blood flow, and increased susceptibility to ischemia (23). Vascular disease and endothelial dysfunction lead to erectile dysfunction through reduced blood inflow, arterial insufficiency or arterial stenosis (24). Previous studies have shown that single variables such as abdominal circumference, BMI, blood pressure and fasting glucose (FPG) and triglycerides (TG) in MetS are promising as predictors of ED (25–27).

Our study found a positive correlation between MetS and ED prevalence, suggesting a potential advantage of MetS in assessing ED prevalence. Currently, the most widely used parameter for assessing obesity is the body mass index, and waist circumference has been shown to be closely related to abdominal obesity as well as visceral fat (28). Studies have demonstrated that genetically predicted body mass index and waist circumference increase the risk of developing ED (29). Obesity affects sex hormone metabolism levels, which is one of the key factors contributing to ED. Corona et al. (30). found that lower androgen levels were a feature of obesity in men with ED after adjusting for comorbidities. Obesity-related comorbidities, especially hypertension, are the most important determinants of arteriogenic obesity-related ED. In obese men, due to the overexpression of aromatase in adipose tissue, obese individuals exhibit increased concentrations of estrogen, which plays an important role in the development of hypogonadism through a negative feedback loop in which men exhibit hypogonadotropic symptoms (31). Factors associated with ED are varied, and obesity is an important component. The METS-VF is a more accurate assessment of obesity than body mass index (BMI). Previous studies have found that age, sex, waist-to-height ratio, and other variables in the METS-VF are expected to serve as an index of intra-abdominal fat content response (32). Our study found a positive correlation between METS-VF and ED prevalence, demonstrating the superiority of METS-VF in assessing ED prevalence. Similarly we emphasized the elevated risk of Aide in men over 40 years of age. Furthermore, we confirmed that METS-VF was superior to WC and BMI in diagnosing ED, emphasizing the robustness in our study results. WWI more accurately differentiates between fat and muscle mass and assesses poor metabolic profiles compared to traditional BMI and waist circumference. Its more longitudinal studies to elucidate the exact causality of these relationships. Waist and hip circumferences are often used to compensate for the fact that BMI does not reflect information about body fat distribution. It is one of the best anthropometric indicators of metabolic syndrome and can be used in the clinical evaluation of metabolic syndrome and obesity, as well as in the prediction of the risk of diabetes and metabolic syndrome. The ABSI combines weight, height, and waist circumference, and provides a more complete picture of an individual’s body size and fat distribution than the BMI. The ABSI was initially developed for use with the U.S. population, and may not be applicable to all races and populations. The ABSI is not intended for use in the United States. The ABSI was originally developed for the U.S. population and may not be applicable to all races and populations. As a relatively new indicator, the ABSI is not as widely recognized and used as the BMI and may not be as popular in some regions or healthcare settings.

Findings from another study suggest that high blood pressure increases the prevalence of ED (33). Others believe that diabetes is another major contributor to ED, increasing the risk of ED onset by 1.3–3 times, even when age and type of diabetes are taken into account (34–36). Bello-Chavolla et al. (37) proposed a fasting score to assess insulin sensitivity, which can be very helpful in assessing the presence of diabetes. Although MetS is not a diagnostic tool for diabetes mellitus or its type, it is effective in reflecting the degree of insulin resistance and has advantages in assessing the adverse consequences of type 2 diabetes mellitus. Moreover, the triglyceride glucose index, calculated from fasting glucose and triglyceride values, has an important role in the diagnosis and follow-up of ED (38). In addition, reactive adipokines produced by abnormal visceral obesity are key drivers of chronic inflammation in the body (25). There is a strong association between inflammation and the development of ED, and the severity of ED is associated with co-morbid conditions, particularly in obese populations (39).

In this study, we conducted the first cross-sectional study of the interaction between the prevalence of metabolic syndrome and ED by utilizing metabolic syndrome-related indicators and combining multiple factors, a methodology that is significant because it has several advantages. In addition, a representative and reliable sample was selected for our study, which further enhances the value of the study. However, there are some limitations of this study that deserve to be recognized. Firstly, there are inherent properties of cross-sectional studies that limit our ability to infer causality. Determining whether a causal relationship exists between MetS and ED and unraveling the unidirectional or bidirectional nature of this association will require further substantiation in a follow-up investigation. Secondly, this study’s assessment of ED relied on participants’ self-report surveys, which is inherently prone to recall bias. Therefore, it is important to conduct prospective follow-up studies to provide more reliable insights. Finally, for ED and MetS, there are multiple potential impacts. Despite considerable efforts to adjust for the inclusion of relevant covariates in our model, completely mitigating the potential impact of other covariates that may play a role remains an ongoing challenge. It is important to conduct well-designed large prospective studies in the future to further substantiate the association between MetS and ED risk.

## 5 Conclusion

By applying data from a representative sample of the U.S. population, we effectively revealed a strong, positive association between MetS and ED prevalence. Mets had a 2.32 times greater risk of ED than non-MetS. Furthermore, this observed positive correlation emphasizes the need for increased vigilance in patients with MetS, smoking, and advanced age.

## Data Availability

The datasets presented in this study can be found in online repositories. The names of the repository/repositories and accession number(s) can be found in the article/supplementary material.
